# Supplementary material for: Elevated Plasma Immunoglobulin Levels Prior to Heart Transplantation Are Associated with Poor Post-Transplantation Survival
Source: Biology (Basel). 2022 Dec 30;12(1):61. doi: 10.3390/biology12010061 (PMC9855413; doi:10.3390/biology12010061)
Supplement: Supplementary file 1 [file biology-12-00061-s001.zip › biology-2042520-supplementary.pdf]

Table S1 – Baseline characteristics of patients with myocardial tissue lysates.

Characteristics	Number	Percentage / STDEV
**Number of patients**	61	
**Sex**		
Male	47	78%
Female	14	23%
**Age** (years)	46	±13
**LVAD pre-HTx**	19	31%
**Survival post-HTx** (years)	6.2	±7.1
**Primary cardiac diagnosis**		
ICM	28	46%
DCM	23	38%
HCM	3	5%
RCM	2	3%
Other	4	7%
**Histological cardiac allograft vasculopathy phenotype**		
H-CAV 0	12	20%
H-CAV 1	8	13%
H-CAV 2	23	37%
H-CAV 3	18	30%

LVAD: left ventricular assist device, HTx: heart transplantation, ICM: idiopathic cardiomyopathy, DCM: dilated cardiomyopathy, HCM: hypertrophic cardiomyopathy, RCM: restrictive cardiomyopathy.

Table S2 – Baseline characteristics of subgroup of patients with available plasma samples

Characteristics	Number	Percentage / STDEV
**Number of patients**	36	
**Sex**		
**Male**	29	81%
**Female**	7	19%
**Age**	46	±14
**LVAD pre-HTx**	14	39%
**Survival post-HTx (years)**	2.6	±3.7
**Primary cardiac diagnosis**		
ICM	15	41%
DCM	13	36%
HCM	2	6%
RCM	2	6%
Other	4	11%
**Histological cardiac allograft vasculopathy phenotype**		
H-CAV 0	10	28%
H-CAV 1	5	14%
H-CAV 2	14	39%
H-CAV 3	7	19%

LVAD: left ventricular assist device, HTx: heart transplantation, ICM: idiopathic cardiomyopathy, DCM: dilated cardiomyopathy, HCM: hypertrophic cardiomyopathy, RCM: restrictive cardiomyopathy.

Figure S1: 1-month post-HTx circulating immunoglobulin levels are associated with survival post-HTx.

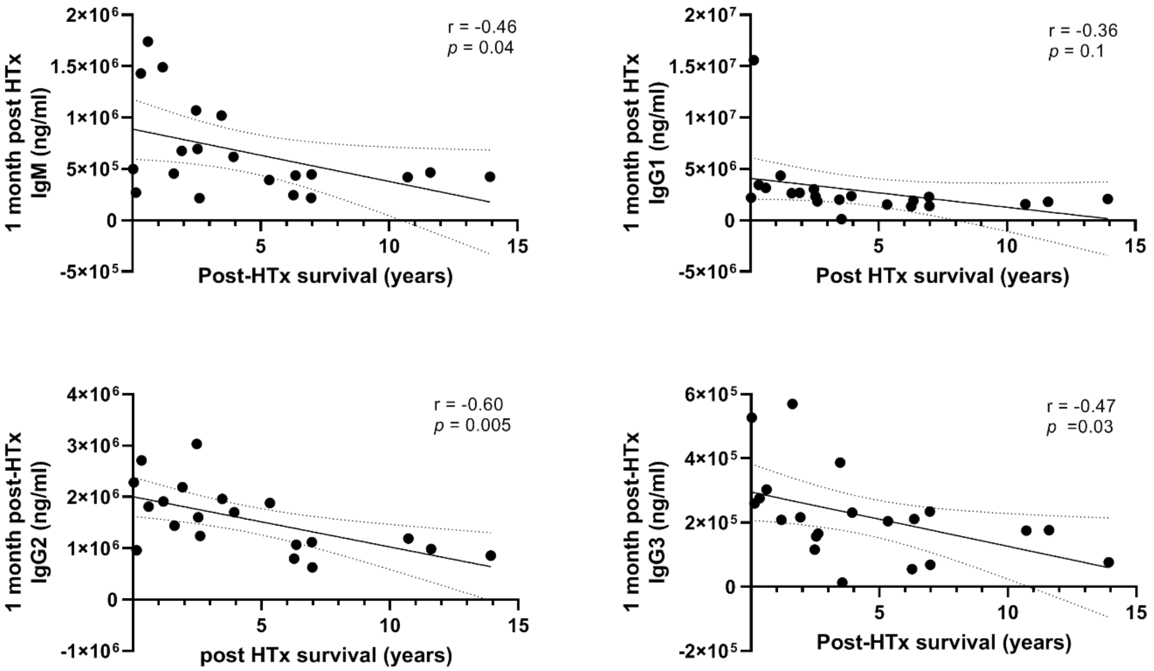

Supplemental Figure S1: In the subgroup of patients with survival of ≥1 month, plasma levels of different immunoglobulin subtypes were measured 1 month post-HTx using multiplex immunoassay and correlated with survival post-HTx. Levels of IgM, IgG2, and IgG3 were significantly correlated with post-HTx survival, where high immunoglobulin levels resulted in a lower survival post-HTx. *HTx: heart transplantation. n=21.*

Figure S2: Plasma levels of immunoglobulins pre- and post-HTx

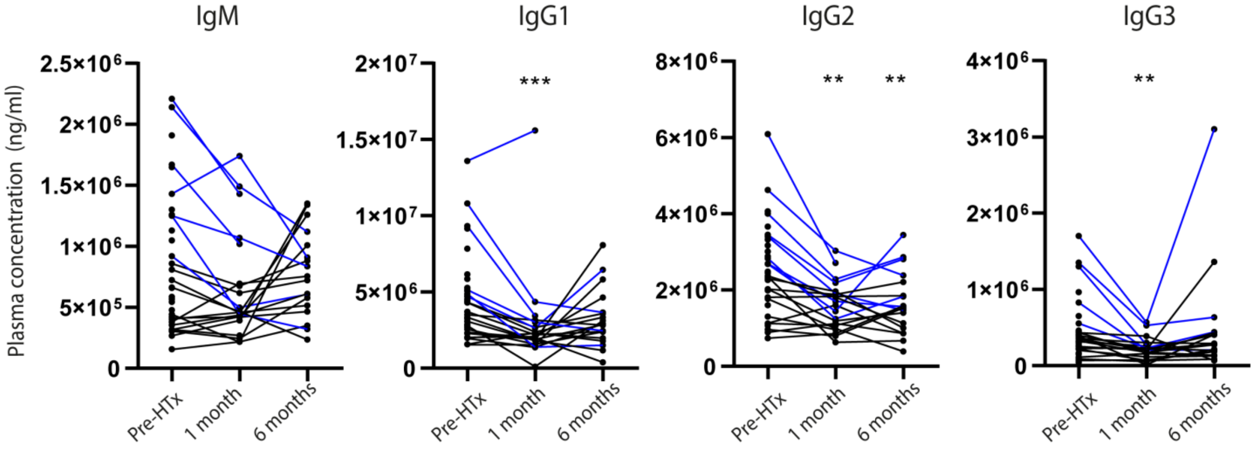

Supplemental Figure S2: The levels of immunoglobulins in plasma were measured prior to HTx until 6 months post transplantation. The blue lines represent patients with high pre-HTx levels (above the mean concentration) and the black lines represent patients with low pre-HTx levels (below the mean concentration). Levels of IgG1, IgG2, and IgG3 were significantly decreased after 1 month of HTx compared to pre-HTx levels. After 6 months, immunoglobulin levels seem to increase again, albeit not statistical significant. *HTx: heart transplantation plasma samples pre-HTx n=36, 1 month n=21, 6 months n=19. ** p<0.01, ** p<0.01.*
